# Using Species-Area Relationships to Inform Baseline Conservation Targets for the Deep North East Atlantic

**DOI:** 10.1371/journal.pone.0058941

**Published:** 2013-03-20

**Authors:** Nicola L. Foster, Andrew Foggo, Kerry L. Howell

**Affiliations:** Marine Biology and Ecology Research Centre, School of Marine Science and Engineering, Plymouth University, Plymouth, United Kingdom; The Australian National University, Australia

## Abstract

Demands on the resources of the deep-sea have increased in recent years. Consequently, the need to create and implement a comprehensive network of Marine Protected Areas (MPAs) to help manage and protect these resources has become a global political priority. Efforts are currently underway to implement MPA networks in the deep North East Atlantic. To ensure these networks are effective, it is essential that baseline information be available to inform the conservation planning process. Using empirical data, we calculated conservation targets for sessile benthic invertebrates in the deep North East Atlantic for consideration during the planning process. We assessed Species-Area Relationships across two depth bands (200–1100 m and 1100–1800 m) and nine substrata. Conservation targets were predicted for each substratum within each depth band using z-values obtained from fitting a power model to the Species-Area Relationships of observed and estimated species richness (Chao1). Results suggest an MPA network incorporating 10% of the North East Atlantic’s deep-sea area would protect approximately 58% and 49% of sessile benthic species for the depth bands 200–1100 m and 1100–1800 m, respectively. Species richness was shown to vary with substratum type indicating that, along with depth, substratum information needs to be incorporated into the conservation planning process to ensure the most effective MPA network is implemented in the deep North East Atlantic.

## Introduction

The deep-sea, one of the largest ecosystems on earth [1], remains the least explored and understood. However, what little we do know suggests that it supports high levels of biodiversity as well as important biological and mineral resources [2], and, thus, warrants effective management to prevent overexploitation and loss of biodiversity. Demands on the resources of deep-sea areas have increased in recent years. As inshore fisheries have become increasingly less productive and advances in technology have created improved gear, more offshore and deeper fisheries have become exploited [2]. In addition, new technology has allowed the exploration of regions of the oceans beyond 2000 m for prospective mineral and hydrocarbon extraction activities [3]. As a result, the need to manage these activities, and protect the resources within the deep-sea has become critical. There are a variety of possible management tools that can be implemented to protect these resources; however, for deep-sea sessile benthic invertebrates Marine Protected Areas (MPAs) are likely to be the most viable option. Here, we respond to a global policy need for data-driven conservation targets to assist in the establishment of MPAs in the deep sea to protect benthic biodiversity.

In recent years, the importance of deep-sea habitats has been recognized both nationally and internationally and efforts are currently underway to implement MPA networks in the deep NE Atlantic [4], as well as the wider High Seas, and deep-sea regions of national territorial waters of Australia, New Zealand, the USA and Canada. However, poor understanding of deep water ecosystems is making environmental management of these areas difficult [3]. Data-based guidelines that can aid managers, policy makers and conservation organizations in planning the location and size of MPA networks in the deep-sea are lacking. In order to ensure effective MPA networks for these areas, it is essential that meaningful data, representative of the substrata and species present in the deep-sea, are used to inform conservation targets.

Conservation targets are an integral part of MPA network design and allow the overall goals of conservation planning, representativeness and persistence to be translated into more quantitative and meaningful targets that can be implemented on the ground [5]. Targets for the conservation of biodiversity can be set for species, habitat types, communities and ecosystems [6], and can be divided into two broad categories; coarse-filter approaches, which set targets for vegetation type or land class; and fine-filter approaches, which incorporate species or population information into the planning process [7].

In the past, many conservation targets have been policy-driven, data-free targets that provided useful starting points but have been heavily criticized [6]. More recently, the need to define data-driven, ecologically relevant methods for setting targets has become increasingly important. One such method is the use of species richness data and Species-Area Relationships (SARs) to provide information for a ‘fine-filter approach’ to setting conservation targets [7], and the use of maximum species richness estimators to further support these relationships [8], [9].

Species richness is a fundamental property of any biological assemblage and is a way to describe community and regional diversity, allowing basic comparisons among sites [8], [9]. One of the earliest diversity patterns to be discovered was the SAR, which describes the cumulative number of species encountered as an increasingly greater area within a habitat type is studied [10]. A number of mathematical models have been used to describe the SAR, but the most widely used is the power relationship, where Species  =  *k*(Area)^z^ [7]. *k* is a scaling factor that can be ignored when using proportions or percentages of species and area [7]; z is the rate at which species are accumulated with increasing area [11].

SARs have been applied to many aspects of ecology, including estimating species richness, predicting species extinctions and defining the appropriate size of protected areas [7], [12]–[18]. Although much of the previous work using SARs has focused on terrestrial systems, SARs are equally applicable to the marine environment [18]. The use of SARs offers a number of benefits for the establishment of protected areas; for example, they are based on the well-established island biogeographic theory [19], provide an estimate of the number of species protected or the area required to protect a given proportion of species, and thus can be used to inform area based targets, can be validated using field data allowing adaptive management, and are repeatable and easy to communicate [6]. However, the value of SARs as predictive tools for reserve design depends on the assumed similarity between the areas used to establish an empirical SAR and the areas that are to be protected [20]. For marine systems, SARs can provide a useful tool for estimating biodiversity and predicting the number of species that are likely to be represented in reserves of varying sizes [18], [20].

Here, a benthic classification scheme for the deep NE Atlantic [4] is combined with substratum-specific SARs for the same region to provide biologically meaningfully conservation targets for use in the development of MPA networks in the deep NE Atlantic region.

## Methodology

### Data Collection

Data were collected from the NE Atlantic during 1 month (August – September) in 2005 aboard the commercial research vessel *Kommandor Jack*, during 2 months (August – October) in 2006 and during 1 month (July) in 2009 aboard the commercial research vessel *MV ‘Franklin’*. During the research cruises, 500 m long video-transects (50 in 2005, 49 in 2006 and 27 in 2009) were conducted across 5 sites; Hatton Bank, George Bligh Bank, Rosemary Bank, Rockall Bank and Anton Dohrn Seamount. As we wished to investigate SARs by substrate type, including bedrock, boulders, cobbles and coral reef, we required a sampling method capable of sampling all terrains and preserving species-substrate relationships. Video is the only method available that is capable of this and, thus, was selected as the sampling method of choice. The use of video and images as a deep-sea sampling technique is not new. Underwater photography was adapted for quantitative biological studies in hard-bottom environments in the early 1970s [21] and later the use of video was developed [22]. Since then the use of video and image data in deep-sea sampling of epibenthic megafauna has grown considerably (see [23] for a review).

The Seatronics drop frame camera system, comprising a 5 megapixel Kongsberg digital stills camera (OE14-208) and an integrated DTS 6000 digital video telemetry system, was deployed from the starboard side of the vessels. In 2005, the video stream from the viewing screen of the digital stills camera provided video data; in 2006 and 2009, separate video (Kongsberg 14–366) and stills cameras were used. Cameras were mounted at an oblique angle (video: 24^o^; stills: 22^o^ from the horizontal) to the seabed to aid in species identification. Sensors monitored depth, altitude and temperature, and an Ultra Short Base Line (USBL) beacon provided accurate (to approximately 1 m) position data for the camera frame.

During the tows, vessel speed was approximately 0.5 knots (min 0.3 and max 0.7 knots). The drop frame was towed in the water column between 1 and 3 m (dependent on substrata and currents) above the seabed (average of 1.8 m across all tows). At the beginning of each tow, starting from when the sea floor became visible, a 2–3 min period was allowed before sampling, to enable the camera to stabilize before commencing the transect. The fields of view of both the stills and video cameras were calibrated using a gridded quadrat of known dimensions. Calibrations were made for ‘on bottom’ (drop frame fully landed on the seabed) and at 1, 2 and 3 m above the seabed to aid in quantitative analysis and particle size discrimination of the substratum.

### Video Analysis

There is a well-documented parabolic pattern of faunal diversity with depth, with a diversity peak at ∼2000 m [1], [24]. In addition, a number of authors have identified faunal boundaries or ‘zones’ along the depth gradient within the deep NE Atlantic [25]–[27]. These zones are in fact regions of lesser faunal change bounded by regions of greater faunal change, however, they serve as a useful means of classifying the deep-sea ecosystem into faunally distinct regions for the purposes of environmental management [4]. In order to provide the most useful information to environmental managers and to go some way to considering the continuous change in diversity with depth, samples from depths between 200–1100 m and 1100–1800 m were analyzed separately [4].

Identification of species from video is difficult and in many cases impossible without physical samples; this is particularly problematic when working in the deep sea where our knowledge of the fauna is more limited. The Rockall Trough is one of the most well studied areas of deep-sea in the world. While we have fairly comprehensive megafaunal species lists for the trawlable grounds and so can be reasonably confident of our ability to identify most soft substrate epibenthic megafauna from video data, the fauna of hard substrate areas is less well known. This is reflected in uncertainty of the taxonomy of a number of deep-sea groups (e.g. corals, sponges). In this study, distinct ‘morphospecies’ were defined, catalogued and used in subsequent video analysis. Morphospecies may correspond to species, genus, family or higher taxonomic levels depending on the group. The morphospecies catalogue is available from the authors upon request. Only those morphospecies that could be consistently identified from up to 3 m off the bottom were included in the analysis. This criterion inevitably resulted in size-selective sampling, with only the conspicuous megafauna recorded. However, all forms of sampling are size-selective, and in the case of trawls and video, species-selective also (with some species more effectively caught within a net or more conspicuous in a video). These short comings of the sampling method mean estimates of diversity will undoubtedly be underestimates. However, video remains the only method for surveying deep-sea hard substrate epibenthic megafauna and the only method that does not integrate results over multiple substrate types, as do trawls. Thus, the use of this technique is fully justified.

Videos were reviewed at slow speed and megafaunal morphospecies [28], [29] were identified and quantified (mobile species were excluded from the analysis). For completeness a full list of morphospecies together with their total abundance by depth band is provided in the supplementary material (Table S1 and S2 in File S1). Substratum types (Table 1) along each transect were visually assigned to one of ten classes using the Wentworth Scale [30] and a modified version of the Folk [31] classification [4]. Analysis of grain size from image data is a rapidly expanding field and automated methods have demonstrated mean grain-size of sieved and imaged sediments correspond to within between 8% and 16% [32]. Here, we used expert interpretation based on a quantified field of view, and anticipate error to be within the margins of automated image analysis methods. Transects were then divided into subsamples. Three datasets were produced from the original transect samples, composed of subsamples of 10 m, 20 m and 30 m linear distance, such that each subsample within a transect corresponded to a single substratum type. Subsamples that overlapped two or more substrata, or where subsamples covered < the specified distance (10 m, 20 m or 30 m), were discarded. These three datasets were reviewed, and the use of a 10 m subsample unit deemed most appropriate as discrete substratum types were effectively sampled at this size.

**Table 1:** Summary of observed (Sobs) and estimated (Chao1) species richness of NE Atlantic fauna from 10 different substrata in two depth bands.

Depth band (m)	Substratum	Area (m^2^)	n*	Sobs	Chao1
200–1100	All Data†	61798	4063	189	209
	Bedrock	3377	222	83	139
	Bedrock with Carbonate Veneer	730	48	73	89
	Gravel (Biogenic – not coral)	1597	105	67	104
	Gravel (Boulders & Cobbles)	7681	505	118	148
	Gravel (Coral Rubble)	4152	273	125	153
	Gravelly Sand (Pebbles)	10860	714	85	115
	Mud	3088	203	10	14
	Sand	24595	1617	99	123
	Sandy Gravel (Biogenic – not coral)	289	19	22	43
	Sandy Gravel (Pebbles & Cobbles)	5430	357	65	87
1100–1800	All Data†	20883	1373	210	249
	Bedrock	5324	350	147	168
	Bedrock with Carbonate Veneer	122	8	32	49
	Gravel (Biogenic – not coral)	472	31	22	30
	Gravel (Boulders & Cobbles)	502	33	46	71
	Gravel (Coral Rubble)	2251	148	117	155
	Gravelly Sand (Pebbles)	3118	205	66	111
	Mud	1795	118	10	19
	Sand	3803	250	60	80
	Sandy Gravel (Pebbles & Cobbles)	3498	230	101	174

* number of 10 m subsamples.

† All data  =  all substrata combined.

As a result of surface wave action the drop frame system used constantly varied between 1 and 3 m height off bottom. Thus, the size of the field of view also constantly varied. The altitude of the camera off bottom was calculated and recorded every second. In order to provide a reasonable estimate of the area of seabed covered by 10 m subsample, the oscillation in camera height off bottom was plotted and the average height off bottom calculated. The average height above the seabed of the camera system was 1.8 m, giving length and area of the camera fields of view of 1.38 m and 2.09 m^2^, respectively. Thus, each 10 m subsample comprised 7.28 ‘fields of view’, covering an approximate area of 15.21 m^2^.

### Species Richness

To compare species richness between the two depth bands and across substrata, differences in the median numbers of recorded species per square meter were compared using Kruskal-Wallis tests (data non-normally distributed; Anderson-Darling test, p < 0.05), with subsequent *post-hoc* pairwise comparisons between substrata conducted using Dunn’s test (significance level p < 0.006, following Bonferroni Correction).

### Species Area Relationships & Calculating Conservation Targets

**Figure 1:**
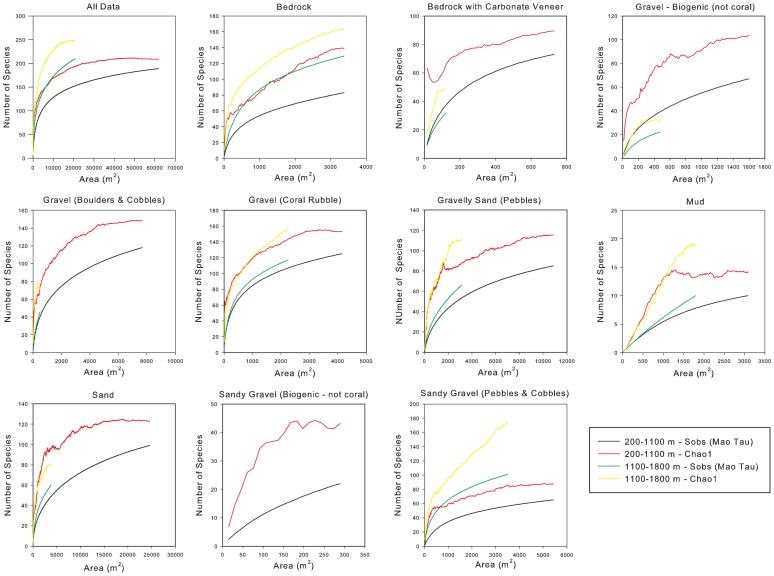
Species accumulation curves for observed (Sobs) and estimated (Chao1) species richness in the NE Atlantic. Species accumulation curves (permuted, 100 runs) are shown for all data combined and per substratum type. Number of samples provided in Table 1 (n).

EstimateS V8.2 [33] was employed to produce a mean species accumulation curve over 100 randomized iterations for the entire dataset and for each substratum type within each depth band. A power model was fitted to each species accumulation curve (SAR) to estimate the z-value (rate of species accumulation [11]) for each dataset (see Table S3 in File S1). Each z-value was then entered into a re-ordered power equation to predict the proportion of species (S) protected for a given area (A) (LogS  =  LogA z) and the proportion of area required to protect a given proportion of species (LogA  =  LogS/z) [7], for all data and for each substratum type. The maximum number of species was not sampled in any substratum type or depth band (species accumulation curves did not reach an asymptote – Fig. 1). A number of techniques have been developed to extrapolate species richness in a discrete assemblage from a limited number of replicate samples [9]. We used EstimateS V8.2 to calculate values of the Chao1 maximum species richness estimator [8], [9] to determine the likely total number of species for each substratum type in each depth band. A power model was then fitted to each of these curves and conservation targets were again calculated as described above.

### Rare and Common Species

Rare species can be defined using a number of different methods [34], [35]. Here, we ranked species according to the number of substrates and sites they were recorded on for both depth bands. The lower quartile of the ranked dataset represented the most restricted (rare) species in terms of their distribution (data not shown). Conservation targets were estimated for both rare and common species independently, as described above.

A range of conservation targets have been recommended for MPA networks in recent years based on various factors (Natural Resource Council [36], [37], [38]). The Convention on Biological Diversity (CBD) has also set out a conservation target of protecting 10% of marine areas by 2020. In order to test the appropriateness of suggested conservation targets for conserving sessile benthic invertebrates in the deep NE Atlantic, we used the widely proposed conservation targets of 10% and 30% of an area in our calculations. In addition, values of 75% and 90% of species were used to represent the majority of diversity in the calculations [7], [39]; high species diversity has been suggested to have a positive influence on ecosystem function and provide a buffer against environmental variations or perturbations [40]–[43].

## Results

### Species Richness

From the two depth bands, all but two substrata were represented by over 30 subsamples (over 450 m^2^ – Table 1), but none of the observed SARs reached an asymptote (Fig. 1), indicating that new species would be encountered with greater numbers of subsamples.

**Figure 2:**
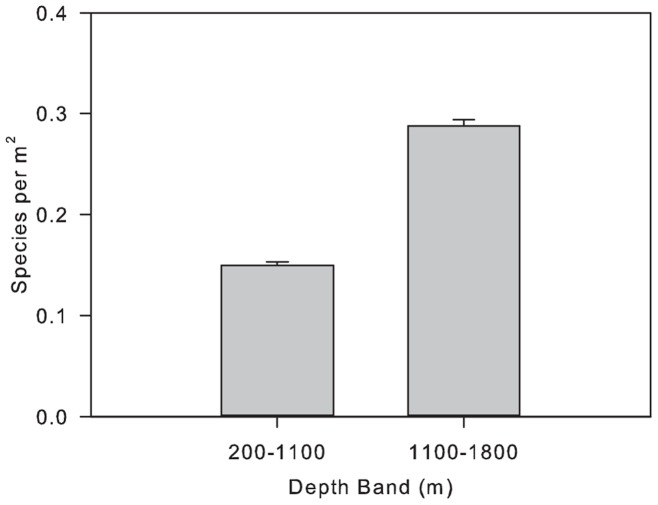
Mean (SE) number of species per m^2^ in two depth bands of the NE Atlantic.

**Figure 3:**
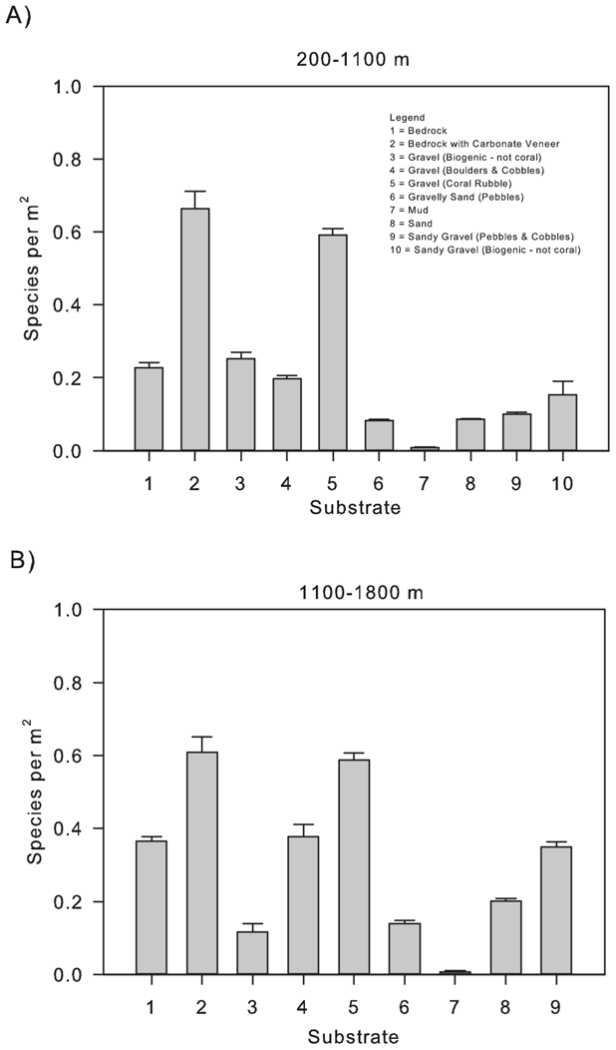
Mean (SE) number of species per m^2^ on different substrata in the NE Atlantic. (A) 200–1100 m depth band and (B) 1100–1800 m depth band.

Species occurred in significantly higher densities at 1100–1800 m compared to 200–1100 m (Kruskal-Wallis H  =  439.96, p < 0.001, df  =  1; Fig. 2). Significant differences were also observed in the number of species per square meter on different substrata (200–1100 m - Kruskal-Wallis H  =  1169.06, p < 0.001, df  =  9; 1100–1800 m – Kruskal-Wallis H  =  584.15, p < 0.001, df  =  8; Fig. 3). Comparison of rank orders of species richness in different substrata based upon observed species with those using Chao1 estimates were consistent, indicating that unsampled species are unlikely to confound the comparisons performed (Table 1).

### Conservation Targets

Conservation targets were calculated for each substratum type within each depth band. Targets calculated using observed species richness (Sobs), irrespective of substratum type, indicate that an MPA network incorporating the widely used conservation target of 10% of the NE Atlantic’s deep-sea area would protect only 58% of species in the 200–1100 m depth band, and only 49% of species in the 1100–1800 m depth band (Table 2). The proportions of species protected increase to 75% and 69% for a conservation target incorporating 30% of the area for the 200–1100 m and 1100–1800 m depth bands, respectively (Table 2).

**Table 2:** Estimates of the percentage of species protected for conservation targets of 10% and 30% in two depth bands of the NE Atlantic.

		Sobs			Chao1		
Depth band (m)	Substratum	z-value	10% Conservation target - species protected (%)	30% Conservation target - species protected (%)	z-value	10% Conservation target - species protected (%)	30% Conservation target - species protected (%)
200–1100	All Data Combined	0.24	58	75	0.16	69	82
	Bedrock	0.39	41	63	0.39	41	63
	Bedrock with Carbonate Veneer	0.39	41	63	0.16	69	82
	Gravel (Biogenic – not coral)	0.47	34	57	0.30	50	70
	Gravel (Boulders & Cobbles)	0.37	43	64	0.25	56	74
	Gravel (Coral Rubble)	0.31	49	69	0.22	60	77
	Gravelly Sand (Pebbles)	0.42	38	60	0.22	60	77
	Mud	0.61	25	48	0.39	41	63
	Sand	0.39	41	63	0.20	63	79
	Sandy Gravel (Biogenic – not coral)	0.67	21	45	0.40	40	62
	Sandy Gravel (Pebbles & Cobbles)	0.40	40	62	0.25	56	74
1100–1800	All Data Combined	0.31	49	69	0.25	56	74
	Bedrock	0.35	45	66	0.27	54	72
	Bedrock with Carbonate Veneer	0.57	27	50	0.49	32	55
	Gravel (Biogenic – not coral)	0.60	25	49	0.47	34	57
	Gravel (Boulders & Cobbles)	0.57	27	50	0.38	42	63
	Gravel (Coral Rubble)	0.35	45	66	0.32	48	68
	Gravelly Sand (Pebbles)	0.53	30	53	0.43	37	60
	Mud	0.86	14	36	0.93	12	33
	Sand	0.44	36	59	0.31	49	69
	Sandy Gravel (Pebbles & Cobbles)	0.38	42	63	0.44	36	59

Note: Estimates calculated using z-values from observed species richness (Sobs) and the Chao1 species richness estimator, and the equation LogS  =  LogA z, where S  =  species and A  =  area [7] . Data are shown for all species.

Using estimated (Chao1) species richness, rather than observed species richness, indicates that the proportion of species protected by an MPA network incorporating 10% of the deep NE Atlantic would be 69% and 56% of species in the 200–1100 m and 1100–1800 m depth bands, respectively, whilst an MPA network incorporating 30% of the area could protect as many as 82% and 74% of species in the two depth bands, respectively (Table 2).

As described above, patterns in species richness were substratum dependent (Fig. 3). Conservation targets calculated for each substratum type separately (using observed species richness) indicated varying proportions of species would be protected for a given area within the two depth bands (Table 2). For example, in the 200–1100 m depth band, a conservation target of 30% of each substratum would protect on average 69% of species on ‘Gravel (Coral Rubble)’ but only 48% of species on ‘Mud’; and 66% of species on ‘Gravel (Coral Rubble)’ but only 36% of species on ‘Mud’ in the 1100–1800 m depth band (Table 3). Predictions made using estimated species richness showed the same pattern with substratum type, though the proportions of species protected were greater (Table 2).

**Table 3:** Estimates of the conservation target size required to protect 75% and 90% of species within two depth bands of the NE Atlantic.

		Sobs			Chao1		
Depth band (m)	Substratum	z-value	75% Species protected – area required (%)	90% species protected – area required (%)	z-value	75% species protected – area required (%)	90% species protected – area required (%)
200–1100	All Data Combined	0.24	30	64	0.16	17	52
	Bedrock	0.39	48	76	0.39	48	76
	Bedrock with Carbonate Veneer	0.39	48	76	0.16	17	52
	Gravel (Biogenic – not coral)	0.47	54	80	0.30	38	70
	Gravel (Boulders & Cobbles)	0.37	46	75	0.25	32	66
	Gravel (Coral Rubble)	0.31	40	71	0.22	27	62
	Gravelly Sand (Pebbles)	0.42	50	78	0.22	27	62
	Mud	0.61	62	84	0.39	48	76
	Sand	0.39	48	76	0.20	24	59
	Sandy Gravel (Biogenic – not coral)	0.67	65	85	0.40	49	77
	Sandy Gravel (Pebbles & Cobbles)	0.40	49	77	0.25	32	66
1100–1800	All Data Combined	0.31	40	71	0.25	32	66
	Bedrock	0.35	44	74	0.27	34	68
	Bedrock with Carbonate Veneer	0.57	60	83	0.49	56	81
	Gravel (Biogenic – not coral)	0.60	62	84	0.47	54	80
	Gravel (Boulders & Cobbles)	0.57	60	83	0.38	47	76
	Gravel (Coral Rubble)	0.35	44	74	0.32	41	72
	Gravelly Sand (Pebbles)	0.53	58	82	0.43	51	78
	Mud	0.86	72	88	0.93	73	89
	Sand	0.44	52	79	0.31	40	71
	Sandy Gravel (Pebbles & Cobbles)	0.38	47	76	0.44	52	79

Note: Estimates calculated using z-values from observed species richness (Sobs) and the Chao1 species richness estimator, and the equation Log A  =  Log S/z, where S  =  species and A  =  area [7]. Data are shown for all species.

The proportion of area required to protect a given percentage of species also varied with substratum type and depth (Table 3). Using observed species richness across all substratum types, 30% and 40% of an area would need to be protected to conserve 75% of species in the 200–1100 m and 1100–1800 m depth bands, respectively. These estimates also varied with substratum type: 40% of a ‘Gravel (Coral Rubble)’ substratum would need to be protected to conserve 75% of species compared to 62% of a ‘Mud’ substratum, in the 200–1100 m depth band (Table 3). Similar patterns were observed when using Chao1 estimated species richness, however, the areas required to protect the given proportion of species were lower (Table 3).

### Rare and Common Species

Estimates of conservation targets using only common species resulted in an increase in the proportion of species protected within a given area and a decrease in the area required to protect a given proportion of species compared to estimates considering all species, using both observed and estimated species richness (see Tables S4 & S5). Expectedly, estimates for rare species alone resulted in a lower proportion of species protected within a given area and an increase in the area required to protect a given proportion of species compared to estimates considering all species, using both observed and estimated species richness (see Tables S4 & S5).

## Discussion

The results of this study provide the first, data-driven conservation targets for use in conservation planning in the deep sea, and could help to improve management of deep-sea sessile benthic species. We demonstrate that the widely proposed conservation target set out by the global Convention on Biological Diversity, to protect 10% of marine areas by 2020, would not be sufficient to conserve the majority of sessile benthic species within the deep NE Atlantic, protecting only 49–58% of observed species, depending on depth (Table 2). High species diversity has been suggested to stabilize ecosystem function and provide a buffer against environmental variation or perturbations [40]–[43]. In order to conserve the majority of species (i.e., 75% of all species [7], [39]) within the deep NE Atlantic, an MPA network would need to incorporate between 30% and 40% (Table 3), depending on depth. It is important to note that the conservation targets provided here are based on inclusion of both common and rare species. Protection of rare species alone would require much larger conservation targets (see Tables S4 & S5), while protection of the more dominant species would require smaller conservation targets (see Tables S4 & S5).

MPAs are widely acknowledged as an important tool for managing human activities in marine ecosystems [44]. A range of conservation targets have been recommended for MPA networks in recent years, depending on the region, the level of human impacts and disturbances, and the rationale behind the network establishment [36]–[38]. However, the general consensus is that maximum benefits are gained when an MPA network protects at least 20–40% of an area (Natural Resource Council [36], [37], [39], [45], [46]). A conservation target of 30% was tested in this study and was predicted to protect 69–75% (74–82% based on Chao1 estimates; Table 2) of sessile benthic invertebrates, depending on depth. This larger conservation target would conserve close to the majority of sessile benthic species (75%) in the deep NE Atlantic, thereby maintaining high species diversity associated with stable ecosystem function [47], [48].

The differences in the estimated conservation targets between depth bands is most likely related to the well documented bathymetric gradient in epibenthic megafaunal species diversity reported from this region. In the NE Atlantic, parabolic patterns of species diversity with depth have been demonstrated for a variety of macrofaunal and megafaunal taxa [1], [49]–[53], with maximum diversity occurring at around 2000 m for the NE Atlantic megafauna [27], [49], [54]. These data suggest that at depths at which maximum diversity occurs, a larger area would need to be protected in order to achieve any given proportion of species protection. The corollary of this is that as diversity decreases below 2000 m a smaller area may be required to achieve the same level of protection at abyssal depths. However, this proposition needs to be tested before any firm conclusions can be drawn. It is possible that changes in substratum type could offset the decrease in area required as a result of a decrease in diversity (see below).

Patterns of change in diversity with depth are not universal in the deep-sea. In their study of gastropod diversity in 10 basins of the Atlantic and Norwegian Sea, Stuart and Rex [24] found no consistent pattern in the diversity gradient with depth, with diversity increasing, decreasing or showing no relationship to depth. They also found a different pattern of diversity for gastropods in the NE Atlantic to previous studies on epibenthic megafaunal taxa. Levels of diversity also varied relative to the NW Atlantic, being depressed in the NE Atlantic and Norwegian Basins, and generally elevated at tropical latitudes and abyssal regions where food supply is high. How applicable the findings of the present study are to other deep-sea areas is therefore unknown. While they are likely to be reasonably reliable for the North Atlantic they may be less applicable to other ocean basins.

Consideration of faunal gradients with depth is important in the context of MPA planning. Although two distinct depth bands are considered here, following Howell (2010), it is important for marine environmental managers to understand that the deep-sea fauna, and the faunal pattern of diversity, changes continuously with depth [27], [55]–[57]. The ‘zones’ or ‘classes’ suggested by Howell (2010) are, in fact, regions of lesser faunal change bounded by regions of greater faunal change. Thus, in order to achieve the 75% of sessile benthic species being protected by ∼ 30% of the area, as identified by this study, the area protected should span the full depth range of the zone under consideration, and ideally should span the full depth range of the continental slope e.g., protected areas within depth ‘zones’ should be continuous with those in adjacent zones.

Species richness varied with substratum type (Figs 1 & 3, Table 1), indicating that, along with depth, substratum information should where possible be incorporated into the conservation planning process to ensure the most effective MPA network is implemented. It is interesting to note that the species rich substratum classes related to cold water coral reefs (Bedrock with Carbonate Veneer; Gravel (coral rubble)) require a much smaller percentage area to conserve the same proportion of species than would be required when considering the more species poor Mud substratum class (Tables 2 and 3). There is a well-established relationship between depth and substratum type whereby with increasing depth the percentage of gravel and exposed bedrock, detrital sand and silts decrease, being replaced by clays and deep-sea oozes [58]. This suggests again that at mid and lower slope depths a greater area may be required in order to conserve a given percentage of species. Contrary to the suggestion above of a smaller area being required at abyssal depths as a result of the decrease in diversity, a larger area may therefore be required as a result of the abyssal plain being composed of clays (included here in the mud class) and oozes, where megafaunal species’ densities are much lower [59]–[61]. Further investigation of SARs at lower slope and abyssal depths is required in order to assess the effect of this potential trade off.

While we have provided the first data-driven predictions of conservation targets for the deep-sea, there are limitations to the study. The most important of which is, although *z*-values provide an indication of the rate at which species are accumulated they lack information concerning where species are located in the landscape, or which particular 30% of the area is required to represent the 75% of species being targeted [7]. We have gone some way to accounting for this problem by creating species accumulation curves for different substratum types across different depth bands to provide some indication as to where the majority of sessile invertebrate diversity is located in the deep NE Atlantic. Nevertheless, species density and distribution in the deep-sea are not homogenous and can vary greatly within a habitat, depth band or substratum. Furthermore, rare species can be vulnerable to exploitation and extirpation, and the results presented here demonstrate that rare species should be given specific consideration during conservation planning. Conversely, shared species can lead to a degree of complementarity among substrata and depths, and a species that inhabits a number of different substrata or depth zones is likely to receive greater benefits from an MPA network than a species that inhabits just a single substratum or depth zone. Thus, conservation target estimates provided here are conservative and substratum type, depth and species occurrence need to be incorporated into conservation planning to ensure that adequate protection is provided to as many species as possible.

Additionally, substratum-specific SARs can be sensitive to data quality and quantity: patchily available data can bias the results towards substrata with more data and towards species with higher densities, more even distribution and hence higher probabilities of being sampled [6]. While every attempt was made to adequately sample each substratum type within the deep NE Atlantic, the very nature of the ecosystem and sampling conditions made this very difficult. The vastness of the ecosystem combined with funding and time constraints mean the majority of deep-sea sampling is conducted along specific targeted features, such as seamounts and canyons, rather than as random transects across the entire region, leading to an inevitable bias in the representation of substrata and depth bands. Within this study, ‘coarse and mixed sediment’ and ‘rock’ substrata were over-represented and ‘mud’ and ‘sandy’ substrata were under-represented. While one option would have been to reduce the number of over-represented substratum samples to match the number of under-represented substratum samples we did not feel that this was appropriate as it would remove a large amount of valuable data from the study in an already data-poor field. Furthermore, the use of the Chao1 species richness estimator accounted for undersampling, and comparisons of rank orders of species richness in different substrata using observed species richness and Chao1 species richness estimates were consistent, indicating that unsampled species are unlikely to confound the comparisons performed.

One of the strengths of using substratum-specific SARs is that they can be updated when additional data becomes available, allowing adaptive management of the MPA network [6]. As research and exploration of the deep NE Atlantic continues, further data can be added to the current study to further validate the predicted conservation targets for sessile benthic invertebrates, and if necessary MPA networks in the region can be adaptively managed to incorporate these changes. Given that the current option for determining conservation targets for the deep-sea is typically to select an arbitrary figure, the use of data-driven conservation targets predicted here can only serve to improve the environmental management of sessile benthic invertebrates in the deep NE Atlantic.

## Acknowledgments

Thanks to Daniel Buscombe for mathematical assistance; Jamie Davies, Charlotte Marshall, Sophie Mowles, Rebecca Ross and Laura Robinson for video analysis and/or data formatting. We would like to thank the partners of the UK Deep-Sea MPA Network Project: H. Stewart (BGS), C. Jacobs (NOCS), N. Golding (JNCC), B. Narayanaswamy (SAMS). Additional thanks to the scientists, officers and crew of RV Kommandor Jack and MV Franklin, and the staff at Geotek and Marin Mätteknik AB.

## Supporting Information

File S1 **Supporting information file includes Tables S1, S2, S3, S4, and S5.** Table S1: List of taxa and total abundance in the depth band 200–1100 m. Table S2: List of taxa and total abundance in the depth band 1100–1800 m. Table S3: Species accumulation z-values for observed (Sobs) and estimated (Chao1) species richness. Table S4: Estimates of the percentage of common and rare species protected for conservation targets of 10% and 30% in two depth bands of the NE Atlantic. Table S5: Estimates of the conservation target size required to protect 75% and 90% of common and rare species within two depth bands of the NE Atlantic.
(DOCX)
