# Supplementary material for: Using Species-Area Relationships to Inform Baseline Conservation Targets for the Deep North East Atlantic
Source: PLoS One. 2013 Mar 20;8(3):e58941. doi: 10.1371/journal.pone.0058941 (PMC3604034; doi:10.1371/journal.pone.0058941)
Supplement: File S1 — Supporting information file includes Tables S1, S2, S3, S4, and S5. Table S1: List of taxa and total abundance in the depth band 200–1100 m. Table S2: List of taxa and total abundance in the depth band 1100–1800 m. Table S3: Species accumulation z-values for observed (Sobs) and estimated (Chao1) species richness. Table S4: Estimates of the percentage of common and rare species protected for conservation targets of 10% and 30% in two depth bands of the NE Atlantic. Table S5: Estimates of the conservation target size required to protect 75% and 90% of common and rare species within two depth bands of the NE Atlantic. (DOCX) [file pone.0058941.s001.docx]

Table S1: List of taxa and total abundance in the depth band 200-1100m

| **Taxon Name** | **Abundance** |
| --- | --- |
| *Cidaris cidaris* | 4413 |
| *Lophelia pertusa* | 2691 |
| *Madrepora oculata* | 2115 |
| *Koehlermetra porrecta* | 1683 |
| *Syringammina fragillissima* | 1661 |
| *Stichopathes* cf. *gravieri* | 1303 |
| *Phelliactis* sp. 1 | 1209 |
| Corallimorphidae sp. 1 | 1063 |
| Porifera massive lobose | 985 |
| Cerianthidae sp. 1 | 874 |
| *Pliobothrus* sp. | 713 |
| *Aphrocallistes* sp. | 534 |
| *Caryophyllia* sp. 2 | 534 |
| *Parastichopus tremulus* | 503 |
| *Calveriosoma fenestratum* | 465 |
| Actiniaria sp. 1 | 438 |
| Porifera green encrusting | 414 |
| *Parantipathes* sp. | 407 |
| Cnidaria sp. 1 | 360 |
| *Phakellia ventilabrum* | 350 |
| Stylasteridae sp. 1 | 348 |
| Ascidiacea sp. 2 | 340 |
| cf. *Antipathella* spp. | 240 |
| Actiniaria sp. 9 | 236 |
| Porifera yellow encrusting | 231 |
| Echinoidea sp. 1 | 219 |
| *Caryophyllia* sp. 3 | 218 |
| Echinus spp. | 216 |
| Ophiuroidea sp. 1 | 210 |
| *Bonellia viridis* | 205 |
| *Anthomastus grandiflorus* | 201 |
| Gorgonacea | 182 |
| Porifera massive globose | 175 |
| Paguridae spp | 169 |
| Cirripedia sp. | 161 |
| *Echinus* sp. 1 | 158 |
| Porifera cup sp. 3 | 150 |
| *Brisingella coronata* / *Brisinga endecacnemos* | 138 |
| Isididae sp. 1 | 131 |
| Porifera white encrusting | 122 |
| Porifera cream encrusting | 119 |
| *Spatangus raschi* | 117 |
| Porifera blue encrusting | 116 |
| *Munida sarsi* | 111 |
| Majidae sp. 2 | 98 |
| Octocorallia sp. 1 | 84 |
| *Axinella infundibuliformis* | 83 |
| Gorgonacea sp. 6 | 83 |
| Porifera massive lobose sp. 8 | 81 |
| *Acanella* sp. 1 | 76 |
| *Callogorgia verticillata* | 74 |
| Porifera massive globose sp. 4 | 74 |
| *Reteporella* sp. 1 | 71 |
| Antipatharia sp. 8 | 70 |
| *Ceramaster/Peltaster/Plinthaster* sp. 1 | 69 |
| *Drifa* sp. 1 | 68 |
| *Munida tenuimana* | 66 |
| *Stichastrella rosea* | 60 |
| Alcyonacea sp. 3 | 59 |
| Antipatharia sp. 9 | 57 |
| Ascidiacea sp. 1 | 52 |
| *Porania pulvillus* | 48 |
| Actiniaria sp. 4 | 46 |
| Porifera spherical sp. 1 | 46 |
| *Henricia sanguinolenta* | 45 |
| Alcyonacea sp. 2 | 42 |
| *Chaceon affinis* | 42 |
| Gorgonacea sp. 2 | 42 |
| Gorgonacea sp. 5 | 42 |
| Porifera | 40 |
| *Leiopathes* sp. 1 | 39 |
| Ophiuroidea sp. 2 | 36 |
| Halcampoididae sp. 1 | 35 |
| *Bolocera tuediae* | 34 |
| Zoanthidea | 33 |
| Alcyonacea sp. 1 | 31 |
| *Paromola cuvieri* | 30 |
| Hydrozoa (bushy) | 29 |
| Actiniaria sp. 17 | 27 |
| *Pheronema carpenteri* | 26 |
| Gersemia sp. 2 | 25 |
| Corallimorphidae sp. 2 | 24 |
| Octocorallia sp. 3 | 23 |
| Porifera orange encrusting | 23 |
| Sagartiidae sp. 1 | 23 |
| Gastroptychus | 21 |
| Gorgonacea sp. 7 | 20 |
| Porifera grey encrusting | 19 |
| Porifera massive lobose sp. 2 | 19 |
| Desmophyllum sp. 1 | 18 |
| Sagartiidae sp. 3 | 18 |
| *Pandalus borealis* | 17 |
| Porifera lamellate | 17 |
| *Anthothela grandiflora* | 16 |
| Echinoidea sp. 3 | 16 |
| Gorgonocephalus sp. 1 | 15 |
| Porifera branching | 14 |
| Porifera spherical sp. 3 | 14 |
| Brachiopoda sp. 1 | 13 |
| *Ceramaster/Peltaster/Plinthaster* sp. 2 | 13 |
| Ophiuroidea sp. 6 | 13 |
| Ophiuroidea sp. 7 | 13 |
| *Bathypathes* sp. 1 | 11 |
| Pennatulacea sp. 1 | 11 |
| Actiniaria | 10 |
| *Asterias rubens* | 10 |
| *Heliometra glacialis* | 10 |
| *Mesothuria intestinalis* | 10 |
| Edwardsiidae sp. 1 | 9 |
| *Epizoanthus* sp. 1 | 9 |
| *Pennatula phosphorea* | 9 |
| Porifera massive globose sp. 9 | 9 |
| Alcyonacea sp. 4 | 8 |
| Aphroditidae | 8 |
| *Colus* sp. 2 | 8 |
| Gastropoda | 8 |
| Porifera branching-erect sp. 1 | 8 |
| Porifera cup | 8 |
| *Stylaster* sp. 1 | 8 |
| Stylasteridae | 8 |
| Actiniaria sp. 8 | 7 |
| Hydrozoa (irregularly branched) | 7 |
| Majidae sp. 1 | 7 |
| Porifera lamellate sp. 4 | 7 |
| *Astropecten irregularis* | 6 |
| Bathynectes sp. | 6 |
| Gorgonacea sp. 3 | 6 |
| Porifera lamellate sp. 6 | 6 |
| *Gersemia* sp. 1 | 5 |
| Porifera massive globose sp. 3 | 5 |
| *Primnoa resedaeformis* | 5 |
| Crinoidea sp. 1 | 4 |
| Geodia sp. 1 | 4 |
| Gorgonacea sp. 1 | 4 |
| Halcampoididae sp. 5 | 4 |
| Porifera cup sp. 2 | 4 |
| Porifera massive lobose sp. 11 | 4 |
| *Virgularia mirabilis* | 4 |
| *Actinauge richardi* | 3 |
| Benthoctopus / Bathypolypus | 3 |
| Bivalvia sp. 1 | 3 |
| Crinoidea sp. 2 | 3 |
| Crinoidea sp. 3 | 3 |
| Echinoidea | 3 |
| *Hippasteria phrygiana* | 3 |
| Octocorallia | 3 |
| Porifera massive globose sp. 7 | 3 |
| Porifera massive lobose sp. 10 | 3 |
| Primnoidae sp. | 3 |
| Sagartiidae sp. 4 | 3 |
| Caridea sp. 1 | 2 |
| Cerianthidae sp. 3 | 2 |
| Cyclostomatida sp. 4 | 2 |
| Decapoda | 2 |
| *Funiculina quadrangularis* | 2 |
| *Kophobelemnon stelliferum* | 2 |
| Margarites sp. 1 | 2 |
| Mysida sp. 2 | 2 |
| Pectinidae spp | 2 |
| Porifera massive lobose sp. 3 | 2 |
| Pterasteridae sp. 1 | 2 |
| Pterasteridae sp. 2 | 2 |
| Sagartiidae sp. 2 | 2 |
| Actiniaria sp. 10 | 1 |
| Actiniaria sp. 11 | 1 |
| Actiniaria sp. 16 | 1 |
| Aeolidiidae sp. 1 | 1 |
| Anthozoa sp. 2 | 1 |
| Ascidiacea | 1 |
| *Benthogone* sp. | 1 |
| *Boreonymphon* sp. | 1 |
| Crinoidea sp. 5 | 1 |
| Cyclostomatida sp. 1 | 1 |
| Decapoda sp. 2 | 1 |
| Decapoda sp. 1 | 1 |
| Halcampoididae sp. 3 | 1 |
| Hydrozoa (flat branched) | 1 |
| Ophiuroidea sp. 3 | 1 |
| Ophiuroidea sp. 8 | 1 |
| *Pachycerianthus multiplicatus* | 1 |
| Pennatulacea sp. 2 | 1 |
| Porifera lamellate sp. 1 | 1 |
| Porifera lamellate sp. 7 | 1 |
| Porifera massive globose sp. 10 | 1 |
| Porifera pink encrusting | 1 |
| Unknown sp. 7 | 1 |

Table S2: List of taxa and total abundance in the depth band 1100-1800m

| **Taxon Name** | **Abundance** |
| --- | --- |
| *Caryophyllia sp. 2* | 5444 |
| *Syringammina fragillissima* | 5169 |
| Porifera blue encrusting | 2071 |
| Porifera green encrusting | 1819 |
| *Lophelia pertusa* | 1616 |
| *Madrepora oculata* | 972 |
| *Phakellia ventilabrum* | 639 |
| Cerianthidae sp. 1 | 587 |
| Porifera massive lobose sp. 18 | 519 |
| Porifera yellow encrusting | 499 |
| Gorgonacea sp. 6 | 431 |
| *Pennatula phosphorea* | 379 |
| Porifera lamellate sp. 10 | 234 |
| *Callogorgia verticillata* | 194 |
| *Koehlermetra porrecta* | 191 |
| *Pheronema carpenteri* | 188 |
| Porifera white encrusting | 172 |
| *Ophiomusium lymani* | 160 |
| Keratoisis sp. 3 | 140 |
| *Callogorgia sp.* | 132 |
| Cirripedia sp. | 127 |
| Porifera massive globose sp. 4 | 124 |
| Porifera grey encrusting | 109 |
| *Pentametrocrinus atlanticus* | 107 |
| *Anthomastus grandiflorus* | 103 |
| Keratoisis sp. 2 | 103 |
| *Calveriosoma fenestratum* | 102 |
| Ophiuroidea sp. 1 | 101 |
| *Halipteris sp.* | 100 |
| Brisingida sp. | 93 |
| Actiniaria sp. 1 | 89 |
| *Aphrocallistes sp.* | 85 |
| *Lepidisis sp.* | 79 |
| Isididae sp. 1 | 78 |
| Porifera massive lobose sp. 6 | 78 |
| Echinus indet. | 71 |
| Actiniaria indet. | 68 |
| Gorgonacea sp. 12 | 65 |
| *Psolus squamatus* | 64 |
| *Asconema setubalense* | 60 |
| *Phelliactis sp.* | 60 |
| Caridea sp. 1 | 57 |
| *Cidaris cidaris* | 51 |
| Porifera cup sp. 3 | 49 |
| Mysida sp. 2 | 48 |
| Velatida sp. 1 | 48 |
| Gorgonacea indet. | 46 |
| Porifera massive lobose sp. 24 | 46 |
| Ophiuroidea sp. 10 | 45 |
| Keratoisis sp. 1 | 41 |
| Actiniaria sp. 4 | 39 |
| *Brisingella coronata / Brisinga endecacnemos* | 38 |
| *Bathypathes sp. 2* | 37 |
| Echinoidea sp. 1 | 37 |
| *Caryophyllia sp. 3* | 35 |
| Antipatharia sp. 6 | 34 |
| *Asterias rubens* | 34 |
| *Stylaster sp. 1* | 32 |
| *Echinus acutus* | 28 |
| Porifera cream encrusting | 27 |
| Actiniaria sp. 18 | 26 |
| *Bonellia viridis* | 26 |
| Porifera spherical sp. 1 | 25 |
| *Anthothela grandiflora* | 24 |
| Porifera boring sp. 1 | 23 |
| Antipatharia sp. 7 | 22 |
| *Actinauge richardi* | 20 |
| *Benthogone sp.* | 20 |
| *Henricia sanguinolenta* | 18 |
| Alcyonacea sp. 1 | 17 |
| Antipatharia sp. 4 | 17 |
| Leiopathes sp. 2 | 17 |
| Porifera branching-erect sp. 1 | 17 |
| Porifera massive globose sp. 9 | 17 |
| Porifera massive lobose sp. 3 | 17 |
| Sagartiidae sp. 1 | 17 |
| Gorgonacea sp. 15 | 16 |
| Porifera lamellate sp. 9 | 15 |
| *Kophobelemnon stelliferum* | 14 |
| Porifera massive globose sp. 3 | 14 |
| Primnoidae sp. | 14 |
| *Stichastrella rosea* | 14 |
| Majidae sp. 2 | 13 |
| Porifera massive lobose sp. 8 | 13 |
| *Solaster endeca* | 13 |
| Alcyonacea sp. 4 | 12 |
| *Heliometra glacialis* | 12 |
| Serpulidae sp. 1 | 12 |
| Antipatharia sp. 9 | 11 |
| *Munida tenuimana* | 11 |
| Porifera cup indet. | 11 |
| Actiniaria sp. 10 | 10 |
| *Caryophyllia sp. 5* | 10 |
| Porifera lamellate sp. 1 | 10 |
| Unknown sp. 25 | 10 |
| Actiniaria sp. 8 | 9 |
| Crinoidea sp. 2 | 9 |
| Holothuroidea sp. 4 | 9 |
| *Parantipathes sp.* | 9 |
| Porifera cup sp. 2 | 9 |
| *Acanella sp. 1* | 8 |
| Holothuroidea sp. 2 | 8 |
| Ophiuroidea sp. 7 | 7 |
| Porifera massive globose sp. 1 | 7 |
| *Leptometra celtica* | 6 |
| Porifera massive lobose sp. 16 | 6 |
| Actiniaria sp. 16 | 5 |
| Crinoidea sp. 1 | 5 |
| *Crossaster papposus* | 5 |
| Gorgonacea sp. 5 | 5 |
| Porifera purple encrusting | 5 |
| *Stichopathes sp.* | 5 |
| *Bolocera tuediae* | 4 |
| *Ceramaster/Peltaster/Plinthaster sp. 1* | 4 |
| Echinoidea sp. 3 | 4 |
| Echinus sp. 1 | 4 |
| *Gastroptychus formosus* | 4 |
| Porifera massive globose sp. 7 | 4 |
| Porifera massive lobose indet. | 4 |
| Porifera massive lobose sp. 2 | 4 |
| Pterasteridae sp. 1 | 4 |
| Sagartiidae sp. 4 | 4 |
| *Astropecten irregularis* | 3 |
| Echinoidea sp. 5 | 3 |
| Echiura sp. 1 | 3 |
| Gorgonacea sp. 1 | 3 |
| Majidae sp. 1 | 3 |
| *Mesothuria intestinalis* | 3 |
| *Munida sarsi* | 3 |
| Mysida sp. 1 | 3 |
| *Porania pulvillus* | 3 |
| Porifera branching-erect sp. 3 | 3 |
| Porifera lamellate sp. 2 | 3 |
| Porifera lamellate sp. 4 | 3 |
| Porifera lamellate sp. 6 | 3 |
| Porifera massive lobose sp. 25 | 3 |
| Porifera massive lobose sp. 5 | 3 |
| Pterasteridae sp. 2 | 3 |
| *Virgularia mirabilis* | 3 |
| *Acanthogorgia granulata* | 2 |
| *Chaceon affinis* | 2 |
| Crinoidea sp. 8 | 2 |
| Crinoidea sp. 9 | 2 |
| Cyclostomatida sp. 4 | 2 |
| Decapoda indet. | 2 |
| Echinoidea sp. 4 | 2 |
| Octocorallia sp. 1 | 2 |
| *Pachycerianthus multiplicatus* | 2 |
| Paguridae indet. | 2 |
| *Paromola cuvieri* | 2 |
| *Placogorgia graciosa* | 2 |
| *Pliobothrus sp.* | 2 |
| Porcellanastor | 2 |
| Porifera cup sp. 3 | 2 |
| Porifera lamellate sp. 11 | 2 |
| Porifera massive lobose sp. 22 | 2 |
| Porifera massive lobose sp. 23 | 2 |
| Porifera orange encrusting | 2 |
| Porifera red encrusting | 2 |
| Porifera spherical sp. 3 | 2 |
| Sagartiidae sp. 3 | 2 |
| *Stichopathes cf. gravieri* | 2 |
| Stylasteridae sp. 1 | 2 |
| Actiniaria sp. 14 | 1 |
| Actiniaria sp. 6 | 1 |
| Annelida sp. 3 | 1 |
| *Anthoptilum grandiflorum* | 1 |
| Asteroidea sp. 1 | 1 |
| *Bathypathes sp. 1* | 1 |
| *Candelabrum sp. 1* | 1 |
| Caryophyllidae sp. 2 | 1 |
| *cf. Antipathella spp.* | 1 |
| *Colus sp. 2* | 1 |
| Corallimorphidae sp. 1 | 1 |
| Crinoidea sp. 7 | 1 |
| Cyclostomatida sp. 3 | 1 |
| *Drifa sp. 1* | 1 |
| Echinoidea indet. | 1 |
| Edwardsiidae sp. 1 | 1 |
| Gastropoda indet. | 1 |
| *Gersemia sp. 2* | 1 |
| *Hippasteria phrygiana* | 1 |
| Hydrozoa (bushy) | 1 |
| *Hypsogastropoda* | 1 |
| Leiopathes sp. 1 | 1 |
| Octocorallia indet. | 1 |
| Ophiuroidea sp. 2 | 1 |
| Ophiuroidea sp. 8 | 1 |
| *Pandalus borealis* | 1 |
| *Parastichopus tremulus* | 1 |
| *Plutonaster bifrons* | 1 |
| Porifera cup sp. 1 | 1 |
| Porifera lamellate sp. 3 | 1 |
| Porifera massive globose indet. | 1 |
| Porifera massive globose sp. 11 | 1 |
| Porifera massive globose sp. 12 | 1 |
| Porifera massive globose sp. 13 | 1 |
| Porifera massive globose sp. 2 | 1 |
| Porifera massive globose sp. 6 | 1 |
| Porifera massive lobose sp. 21 | 1 |
| *Pseudarchaster sp. 1* | 1 |
| *Reteporella sp. 1* | 1 |
| *Spatangus raschi* | 1 |
| *Umbellula sp.* | 1 |
| Unknown sp. 30 | 1 |
| Unknown sp. 31 | 1 |
| *Desmophyllum sp. 1* | 0 |
| Unknown sp. 29 | 0 |

Table S3: Species accumulation z-values for observed (Sobs) and estimated (Chao1) species richness in two depth bands of the NE Atlantic.

| **Depth band (m)** | **Substratum** | **Sobs** | | | | **Chao1** | | | |
| --- | --- | --- | --- | --- | --- | --- | --- | --- | --- |
|  |  | **z-value** | **R^2^** | **F** | **p-value** | **z-value** | **R^2^** | **F** | **p-value** |
| 200-1100 | All Data | 0.24 | 0.98 | 212135.0 | <0.0001 | 0.16 | 0.94 | 66201.5 | <0.0001 |
|  | Bedrock | 0.39 | 0.99 | 31555.0 | <0.0001 | 0.39 | 0.98 | 11356.9 | <0.0001 |
|  | Bedrock with Carbonate Veneer | 0.39 | 0.99 | 3243.6 | <0.0001 | 0.16 | 0.91 | 473.4 | <0.0001 |
|  | Gravel (Biogenic – not coral) | 0.47 | 1.00 | 29718.2 | <0.0001 | 0.30 | 0.97 | 3487.2 | <0.0001 |
|  | Gravel (Boulders & Cobbles) | 0.37 | 0.99 | 69182.3 | <0.0001 | 0.25 | 0.97 | 17814.6 | <0.0001 |
|  | Gravel (Coral Rubble) | 0.31 | 0.99 | 24525.2 | <0.0001 | 0.22 | 0.98 | 16557.9 | <0.0001 |
|  | Gravelly Sand (Pebbles) | 0.42 | 0.99 | 89676.5 | <0.0001 | 0.25 | 0.97 | 21233.0 | <0.0001 |
|  | Mud | 0.61 | 0.99 | 18582.9 | <0.0001 | 0.39 | 0.80 | 827.5 | <0.0001 |
|  | Sand | 0.39 | 1.00 | 592776.0 | <0.0001 | 0.20 | 0.92 | 17358.1 | <0.0001 |
|  | Sandy Gravel (Biogenic – not coral) | 0.67 | 1.00 | 6098.8 | <0.0001 | 0.40 | 0.87 | 121.2 | <0.0001 |
|  | Sandy Gravel (Pebbles & Cobbles) | 0.40 | 0.99 | 30396.7 | <0.0001 | 0.25 | 0.97 | 9663.2 | <0.0001 |
| 1100-1800 | All Data | 0.31 | 0.99 | 196694.8 | <0.0001 | 0.25 | 0.96 | 37026.5 | <0.0001 |
|  | Bedrock | 0.35 | 0.99 | 27702.8 | <0.0001 | 0.27 | 0.97 | 12766.6 | <0.0001 |
|  | Bedrock with Carbonate Veneer | 0.57 | 1.00 | 3372.8 | <0.0001 | 0.49 | 0.92 | 81.1 | 0.0001 |
|  | Gravel (Biogenic – not coral) | 0.60 | 0.99 | 3661.2 | <0.0001 | 0.47 | 0.90 | 259.3 | <0.0001 |
|  | Gravel (Boulders & Cobbles) | 0.57 | 1.00 | 92708.1 | <0.0001 | 0.38 | 0.85 | 180.1 | <0.0001 |
|  | Gravel (Coral Rubble) | 0.35 | 0.99 | 10916.5 | <0.0001 | 0.32 | 0.99 | 23583.2 | <0.0001 |
|  | Gravelly Sand (Pebbles) | 0.53 | 1.00 | 144172.6 | <0.0001 | 0.43 | 0.98 | 12309.6 | <0.0001 |
|  | Mud | 0.86 | 1.00 | 207818.9 | <0.0001 | 0.93 | 0.99 | 9706.7 | <0.0001 |
|  | Sand | 0.44 | 1.00 | 81727.4 | <0.0001 | 0.31 | 0.98 | 9757.2 | <0.0001 |
|  | Sandy Gravel (Pebbles & Cobbles) | 0.38 | 0.99 | 38586.9 | <0.0001 | 0.44 | 0.99 | 17186.7 | <0.0001 |

Note: Associated R^2^ values and ANOVA results for all data combined and each substratum type are provided.

Table S4: Estimates of the percentage of species protected for conservation targets of 10% and 30% in two depth bands of the NE Atlantic calculated using z-values from (A) observed species richness (Sobs) and (B) the Chao1 species richness estimator.

|  | | **Sobs – Common Species Only** | | | **Sobs – Rare Species Only** | | |
| --- | --- | --- | --- | --- | --- | --- | --- |
| **Depth band (m)** | **Substratum** | **z-value** | **10% CT - species protected (%)** | **30% CT - species protected (%)** | **z-value** | **10% CT - species protected (%)** | **30% CT - species protected (%)** |
| 200-1100 | All Data Combined | 0.17 | 68 | 81 | 0.76 | 17 | 40 |
|  | Bedrock | 0.39 | 41 | 63 | - | - | - |
|  | Bedrock with Carbonate Veneer | 0.38 | 42 | 63 | 1.00 | 10 | 30 |
|  | Gravel (Biogenic – not coral) | 0.45 | 35 | 58 | 0.73 | 19 | 42 |
|  | Gravel (Boulders & Cobbles) | 0.34 | 46 | 66 | 0.86 | 14 | 36 |
|  | Gravel (Coral Rubble) | 0.29 | 51 | 71 | 1.00 | 10 | 30 |
|  | Gravelly Sand (Pebbles) | 0.41 | 39 | 61 | 0.64 | 23 | 46 |
|  | Mud | 0.61 | 25 | 48 | - | - | - |
|  | Sand | 0.36 | 44 | 65 | 0.63 | 23 | 47 |
|  | Sandy Gravel (Biogenic – not coral) | 0.67 | 21 | 45 | - | - | - |
|  | Sandy Gravel (Pebbles & Cobbles) | 0.40 | 40 | 62 | 1.00 | 10 | 30 |
| 1100-1800 | All Data Combined | 0.23 | 59 | 76 | 0.80 | 16 | 38 |
|  | Bedrock | 0.30 | 50 | 70 | 0.86 | 14 | 36 |
|  | Bedrock with Carbonate Veneer | 0.57 | 27 | 50 | - | - | - |
|  | Gravel (Biogenic – not coral) | 0.59 | 26 | 49 | 0.63 | 23 | 47 |
|  | Gravel (Boulders & Cobbles) | 0.55 | 28 | 52 | 0.87 | 13 | 35 |
|  | Gravel (Coral Rubble) | 0.32 | 48 | 68 | 0.81 | 15 | 38 |
|  | Gravelly Sand (Pebbles) | 0.52 | 30 | 53 | - | - | - |
|  | Mud | 0.86 | 14 | 36 | - | - | - |
|  | Sand | 0.42 | 38 | 60 | 0.72 | 19 | 42 |
|  | Sandy Gravel (Pebbles & Cobbles) | 0.37 | 43 | 64 | 0.79 | 16 | 39 |

(A)

(B)

|  | | **Chao1 – Common Species Only** | | | **Chao1 – Rare Species Only** | | |
| --- | --- | --- | --- | --- | --- | --- | --- |
| **Depth band (m)** | **Substratum** | **z-value** | **10% CT - species protected (%)** | **30% CT - species protected (%)** | **z-value** | **10% CT - species protected (%)** | **30% CT - species protected (%)** |
| 200-1100 | All Data Combined | 0.07 | 85 | 92 | 0.31 | 49 | 69 |
|  | Bedrock | 0.40 | 40 | 62 | - | - | - |
|  | Bedrock with Carbonate Veneer | 0.10 | 79 | 89 | 1.49 | 3 | 17 |
|  | Gravel (Biogenic – not coral) | 0.31 | 49 | 69 | 0.61 | 25 | 48 |
|  | Gravel (Boulders & Cobbles) | 0.20 | 63 | 79 | 0.98 | 10 | 31 |
|  | Gravel (Coral Rubble) | 0.17 | 68 | 81 | 1.86 | 1 | 11 |
|  | Gravelly Sand (Pebbles) | 0.27 | 54 | 72 | 0.61 | 25 | 48 |
|  | Mud | 0.44 | 36 | 59 | - | - | - |
|  | Sand | 0.21 | 62 | 78 | 0.47 | 34 | 57 |
|  | Sandy Gravel (Biogenic – not coral) | 0.36 | 44 | 65 | - | - | - |
|  | Sandy Gravel (Pebbles & Cobbles) | 0.25 | 56 | 74 | 0.97 | 11 | 31 |
| 1100-1800 | All Data Combined | 0.15 | 71 | 83 | 0.58 | 26 | 50 |
|  | Bedrock | 0.20 | 63 | 79 | 1.015 | 10 | 29 |
|  | Bedrock with Carbonate Veneer | 0.46 | 35 | 57 | - | - | - |
|  | Gravel (Biogenic – not coral) | 0.54 | 29 | 52 | 0.58 | 26 | 50 |
|  | Gravel (Boulders & Cobbles) | 0.39 | 41 | 63 | 0.91 | 12 | 33 |
|  | Gravel (Coral Rubble) | 0.28 | 52 | 71 | 1.061 | 9 | 28 |
|  | Gravelly Sand (Pebbles) | 0.41 | 39 | 61 | - | - | - |
|  | Mud | 0.93 | 12 | 33 | - | - | - |
|  | Sand | 0.32 | 48 | 68 | 0.83 | 15 | 37 |
|  | Sandy Gravel (Pebbles & Cobbles) | 0.40 | 40 | 62 | 0.89 | 13 | 34 |

Note: Estimates calculated using z-values from (a) observed species richness (Sobs) and (b) the Chao1 species richness estimator, and the equation LogS = LogA z, where S = species and A = area ([Desmet & Cowling 2004](#_ENREF_1)). Data are shown for common species only and rare species only. Rare species denotes 25% most restricted species in terms of their distribution across sites and substrates. – denotes no species recorded on that substrate type; CT denotes conservation target.

Table S5: Estimates of the conservation target size required to protect 75% and 90% of species within two depth bands of the NE Atlantic calculated using z-values from (A) observed species richness (Sobs) and (B) the Chao1 species richness estimator.

|  | | **Sobs – Common Species Only** | | | **Sobs – Rare Species Only** | | |
| --- | --- | --- | --- | --- | --- | --- | --- |
| **Depth band (m)** | **Substratum** | **z-value** | **75% Species protected –**  **area required (%)** | **90% species protected –**  **area required (%)** | **z-value** | **75% Species protected –**  **area required (%)** | **90% species protected –**  **area required (%)** |
| 200-1100 | All Data Combined | 0.17 | 18 | 54 | 0.76 | 68 | 87 |
|  | Bedrock | 0.39 | 48 | 76 | - | - | - |
|  | Bedrock with Carbonate Veneer | 0.38 | 47 | 76 | 1.00 | 75 | 90 |
|  | Gravel (Biogenic – not coral) | 0.45 | 53 | 79 | 0.73 | 67 | 87 |
|  | Gravel (Boulders & Cobbles) | 0.34 | 43 | 73 | 0.86 | 72 | 88 |
|  | Gravel (Coral Rubble) | 0.29 | 37 | 70 | 1.00 | 75 | 90 |
|  | Gravelly Sand (Pebbles) | 0.41 | 50 | 77 | 0.64 | 64 | 85 |
|  | Mud | 0.61 | 62 | 84 | - | - | - |
|  | Sand | 0.36 | 45 | 75 | 0.63 | 63 | 85 |
|  | Sandy Gravel (Biogenic – not coral) | 0.67 | 65 | 85 | - | - | - |
|  | Sandy Gravel (Pebbles & Cobbles) | 0.40 | 49 | 77 | 1.00 | 75 | 90 |
| 1100-1800 | All Data Combined | 0.23 | 59 | 76 | 0.80 | 70 | 88 |
|  | Bedrock | 0.30 | 50 | 70 | 0.86 | 72 | 88 |
|  | Bedrock with Carbonate Veneer | 0.57 | 27 | 50 | - | - | - |
|  | Gravel (Biogenic – not coral) | 0.59 | 26 | 49 | 0.63 | 63 | 85 |
|  | Gravel (Boulders & Cobbles) | 0.55 | 28 | 52 | 0.87 | 72 | 89 |
|  | Gravel (Coral Rubble) | 0.32 | 48 | 68 | 0.81 | 70 | 88 |
|  | Gravelly Sand (Pebbles) | 0.52 | 30 | 53 | - | - | - |
|  | Mud | 0.86 | 14 | 36 | - | - | - |
|  | Sand | 0.42 | 38 | 60 | 0.72 | 67 | 86 |
|  | Sandy Gravel (Pebbles & Cobbles) | 0.37 | 43 | 64 | 0.79 | 69 | 88 |

(A)

(B)

|  | | **Chao1 – Common Species Only** | | | **Chao1 – Rare Species Only** | | |
| --- | --- | --- | --- | --- | --- | --- | --- |
| **Depth band (m)** | **Substratum** | **z-value** | **75% Species protected –**  **area required (%)** | **90% species protected –**  **area required (%)** | **z-value** | **75% Species protected –**  **area required (%)** | **90% species protected –**  **area required (%)** |
| 200-1100 | All Data Combined | 0.07 | 2 | 22 | 0.31 | 40 | 71 |
|  | Bedrock | 0.40 | 49 | 77 | - | - | - |
|  | Bedrock with Carbonate Veneer | 0.10 | 6 | 35 | 1.49 | 82 | 93 |
|  | Gravel (Biogenic – not coral) | 0.31 | 40 | 71 | 0.61 | 62 | 84 |
|  | Gravel (Boulders & Cobbles) | 0.20 | 24 | 59 | 0.98 | 75 | 90 |
|  | Gravel (Coral Rubble) | 0.17 | 18 | 54 | 1.86 | 86 | 94 |
|  | Gravelly Sand (Pebbles) | 0.27 | 34 | 68 | 0.61 | 62 | 84 |
|  | Mud | 0.44 | 52 | 79 | - | - | - |
|  | Sand | 0.21 | 25 | 61 | 0.47 | 54 | 80 |
|  | Sandy Gravel (Biogenic – not coral) | 0.36 | 45 | 75 | - | - | - |
|  | Sandy Gravel (Pebbles & Cobbles) | 0.25 | 32 | 66 | 0.97 | 74 | 90 |
| 1100-1800 | All Data Combined | 0.15 | 71 | 83 | 0.58 | 61 | 83 |
|  | Bedrock | 0.20 | 63 | 79 | 1.015 | 75 | 90 |
|  | Bedrock with Carbonate Veneer | 0.46 | 35 | 57 | - | - | - |
|  | Gravel (Biogenic – not coral) | 0.54 | 29 | 52 | 0.58 | 61 | 83 |
|  | Gravel (Boulders & Cobbles) | 0.39 | 41 | 63 | 0.91 | 73 | 89 |
|  | Gravel (Coral Rubble) | 0.28 | 52 | 71 | 1.061 | 76 | 91 |
|  | Gravelly Sand (Pebbles) | 0.41 | 39 | 61 | - | - | - |
|  | Mud | 0.93 | 12 | 33 | - | - | - |
|  | Sand | 0.32 | 48 | 68 | 0.83 | 71 | 88 |
|  | Sandy Gravel (Pebbles & Cobbles) | 0.40 | 40 | 62 | 0.89 | 72 | 89 |

Note: Estimates calculated using z-values from (a) observed species richness (Sobs) and (b) the Chao1 species richness estimator, and the equation Log A = Log S/z, where S = species and A = area ([Desmet & Cowling 2004](#_ENREF_1)). Data are shown for common species only and rare species only. Rare species denotes 25% most restricted species in terms of their distribution across sites and substrates. – denotes no species recorded on that substrate type; CT denotes conservation target.

**References**

Desmet, P. & Cowling, R. (2004) Using the species-area relationship to set baseline targets for conservation. *Ecology and Society,* **9**.
